# Patient demographics and MRI-based measurements predict redundant nerve roots in lumbar spinal stenosis: a retrospective database cohort comparison

**DOI:** 10.1186/s12891-018-2364-4

**Published:** 2018-12-22

**Authors:** Luca Papavero, Carlos J. Marques, Jens Lohmann, Thies Fitting

**Affiliations:** 1 Clinic for Spine Surgery, Schoen Clinic Hamburg Eilbek, Dehnhaide 120, D-22081 Hamburg, Germany; 2 Science Office of the Orthopedic and Joint Replacement Department, Schoen Clinic Hamburg Eilbek, Dehnhaide 120, D-22081 Hamburg, Germany; 3 Department of Radiology, Schoen Clinic Hamburg Eilbek, Dehnhaide 120, D-22081 Hamburg, Germany; 4 Non-Medical PhD Program, Faculty of Medicine (UKE), University of Hamburg, Hamburg, Germany

**Keywords:** Redundant nerve roots, Lumbar spinal stenosis, Classification of lumbar spinal stenosis, Length of lumbar spine, Cauda equina claudication

## Abstract

**Background:**

Up to 40% of patients diagnosed with lumbar spinal stenosis (LSS) show evidence of redundant nerve roots (RNR) of the cauda equina on their magnetic resonance images (MRI). The etiology of RNR is still unclear. Preoperative evidence of RNR is associated with a worse postsurgical outcome. Consequently, potential predictors of RNR could have a prognostic value. The aim was to test whether patient demographics and MRI-based measurements can predict RNR in LSS patients.

**Methods:**

In a retrospective database-based cohort study the preoperative data of 300 patients, 150 with (RNR+) and 150 without (RNR-) evidence of RNR on their MRI were analyzed. Three independent researchers performed the MRI reads. Potential predictors were age, gender, body height (BH), length of lumbar spine (LLS), segmental length of lumbar spine (SLLS), lumbar spine alignment deviation (LSAD), relative LLS (rLLS), relative SLLS (rSLLS), number of stenotic levels (LSS-level), and grade of LSS severity (LLS-grade, increasing from A to D). Binomial logistic regression models were performed.

**Results:**

RNR+ patients were 2.6 years older (*p* = 0.01). Weak RNR+ predictors were a two-years age increase (OR 1.06; *p* = 0.02), 3 cm BH decrease (OR 1.09; *p* = 0.01) and a 5 mm SLLS decrease (OR 1.34; *p* < 0.001). Strong RNR+ predictors were a 1% rLLS decrease (OR 2.17; *p* < 0.001), LSS-level ≥ 2 (OR 2.59; *p* = 0.001), LLS-grade C (OR 5.86; *p* = 0.02) and LLS-grade D (OR 18.4; *p* < 0.001). The mean rSLLS of RNR+ patients was 0.6% shorter (*p* < 0.001; 95% C.I. 0.4 to 0.8) indicating a disproportionate shorter lumbar spine.

**Conclusions:**

We identified LSS severity grade and LSS levels as the strongest predictors of RNR. In addition to previous studies, we conclude that a shortened lumbar spine by degeneration is involved in the development of RNR.

## Background

Lumbar spinal stenosis (LSS) is the most common reason for lumbar spine surgery in patients older than 65 years [1]. Around 40% of all LSS patients scheduled for decompression surgery show evidence of RNRs of the cauda equina on their preoperative MRI [2–4].

RNRs were described as thickened, buckling or coiled nerve roots that typically assume serpentine or loop-shape in T2-weighted MR images [5]. When the standard T2-weighted sequence is equivocal, adding a single slice MRI-myelography sequence may help to identify RNRs. RNRs were mostly observed above the stenotic level, but can also be found below, or both above and below the stenotic level [6, 7].

Reports indicated that LSS patients with preoperative evidence of RNRs (RNR+) have a significantly longer mean duration of neurological symptoms and experience less improvement in their ability to walk after surgery in comparison to patients without RNRs (RNR-) [2, 6, 8, 9].

The etiology and pathogenesis of RNRs are still unclear. RNRs seem to be a negative prognostic factor in LSS patients. Therefore, the investigation of factors that may predict the presence of RNRs is of clinical importance. The present study aimed to investigate whether patients’ demographics and MRI-based measurements can predict RNRs in patients scheduled for LLS decompression surgery.

## Methods

### Study design and sample

This is a retrospective database-based cohort comparison study. Reporting of the present study follows the STROBE Statement guidelines for reporting observational studies [10]. The inclusion criteria were symptomatic lumbar spinal canal stenosis requiring surgical decompression without fixation and availability of preoperative MRI that were performed in a scanner with at least 1.5 Tesla, including sagittal T1- and T2-weighted images and axial T2-weighted images in the picture archive and communication system (PACS) of the institution. Exclusion criteria were previous history of lumbar spine surgery, lumbar deformity as scoliosis or vertebral slip requiring fixation and congenital, traumatic, infectious or neoplastic diseases of the lumbar spine.

Sample size was calculated with the use of G*Power version 3.1.9.2 (Psychology Department, Duesseldorf University, Germany) [11]. For sample size calculation the variable LSS-level was chosen and the following assumptions were used: 68% of RNR+ patients show one stenotic level and 32% show two or more stenotic levels; oppositely 84% of RNR- patients show one stenotic level and 16% show two or more stenotic levels. Based on these assumptions an odds ratio of 2.47 was calculated. Thereby, if α = 0.05 and 1-ß error probability = 0.90, there is a 90% chance of correctly rejecting the null hypothesis that a particular value of the main predictor variable (LSS-Level) is not associated with the outcome variable, with a total sample size of 300 patients (150 per group).

The preoperative data of 300 consecutive LSS patients who underwent single- or multi-level microsurgical bilateral decompression via a unilateral approach (also known as “cross over” or “over the top” technique) without any fixation were evaluated. The surgeries were performed by six different surgeons with a level of experience ranging from 4 to 35 years. The ipsilateral facet was resected one third and the contralateral was left alone whereas the thickened yellow ligament was completely removed. The surgeries were performed between December 2012 and August 2016 at the same institution. During this time window 2273 patients underwent decompression surgery for LSS. Thereof 2113 underwent decompression surgery without fixation. Out of this second group patients with and without RNR on their preoperative MRIs were selected from August 2016 backwards, until both groups each contained 150 patients.

The Ethics Committee of the Federal State of Hamburg deliberated upon the research proposal of the present study (File PV5817). According to the ethics committee retrospective database-based studies do not require an approval, whenever the data was acquired, saved and treated anonymously. This applies to the present study.

The database used for this research is not publicly available, it is property of the Schoen Clinic Group, whose access is regulated by the rules of procedure of its in-house Science Office.

The following patient-related and MRI-based factors were used as potential predictors: age, gender, body height (BH), length of the lumbar spine (LLS), segmental length of the lumbar spine (SLLS), relative LLS (rLLS), relative SLLS (rSLLS), the amount of lumbar spine alignment deviation (LSAD), as given by the difference between SLLS and LLS, the number of stenotic levels involved (LSS-level) and the grade of severity of the stenosis (LSS-grade) on a progressive scale from A to D [12].

Firstly, the 300 patients were assigned to either the RNR+ or the RNR- group by a senior radiologist, a senior orthopedic surgeon and a senior neurosurgeon independently. Their experience levels were 15, 10 and 35 years respectively.

**Fig. 1:**
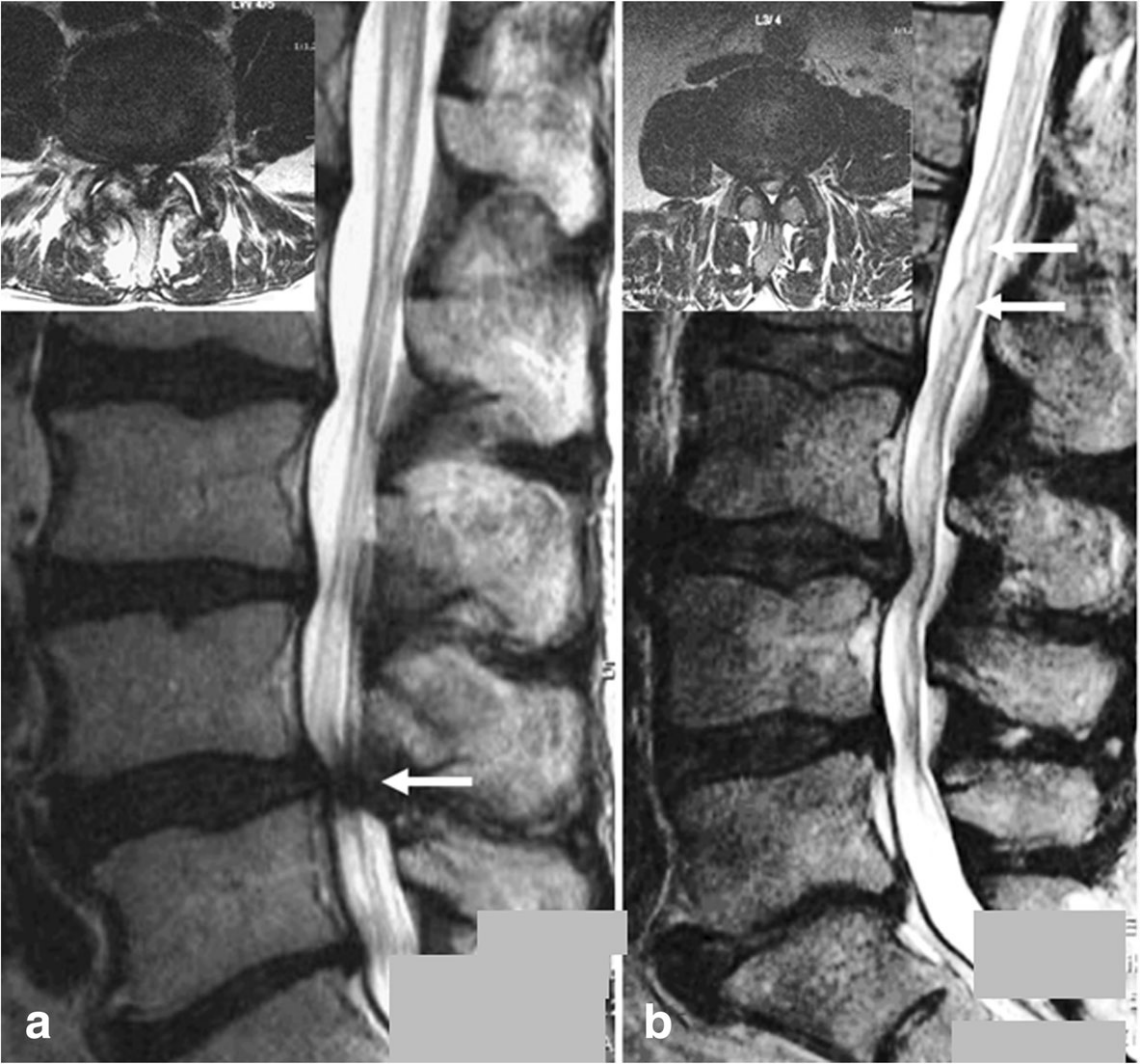
Sagittal T2-WI of spinal canal stenosis (**a**) without and (**b**) with evidence of RNR **a**) Sagittal T2-WI of spinal canal stenosis L4/L5 (axial: upper corner left) with no evidence of RNR since the spatial distribution of the cauda nerve roots is not influenced by the stenotic level (white arrow). **b**) Sagittal T2-WI of spinal canal stenosis L3/L4 (axial: upper corner left) with evidence of serpentine-shaped RNR deflection (white arrows)

**Fig. 2:**
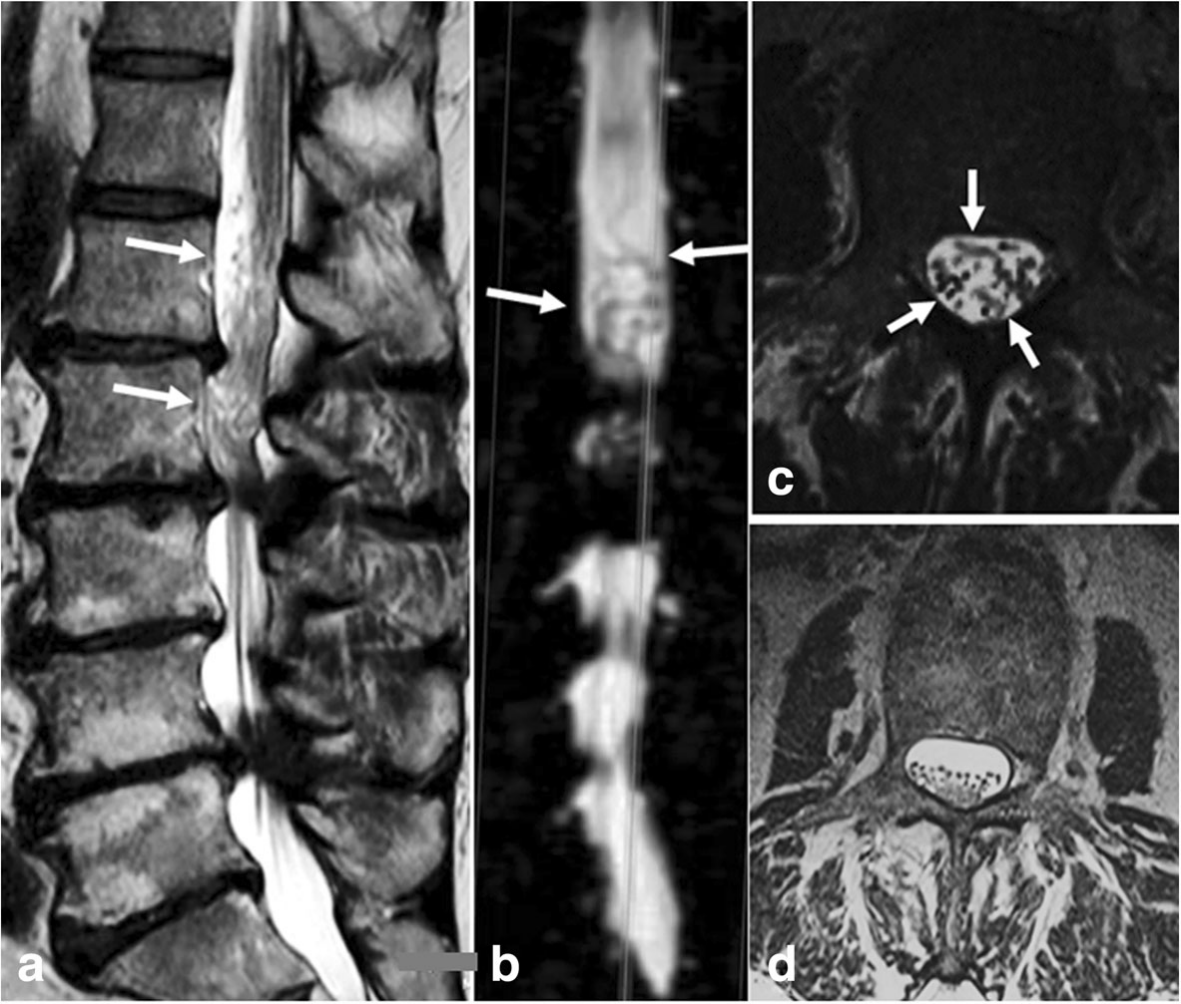
Sagittal T2-WI of spinal canal stenosis with evidence of loop-shaped RNR **a**) Sagittal T2-WI of spinal canal stenosis L2/L3. At this level caudal straightened nerve roots switch to loop like RNR (white arrows); **b**) The coronal Myelo-MRI shows the horizontalized loops (white arrows); **c**) Axial T2-WI shows segments of the loops (white arrows); **d**) Axial T2-WI of a normal lumbar spinal canal: the cauda roots appear as dots

The definition of RNR used to assign the patients into the groups was the following: RNR were defined as serpentines [13] when in sagittal T2-WI a sinusoidal deflection (complete crest-trough wave) occurred within the height of a vertebral body without any horizontalization of the involved roots (Fig. 1b). RNR were defined as loops when in sagittal T2-WI at least in two different areas dots or horizontalized roots (Fig. 2a) were combined with tortuous roots in the axial T2-WI (Fig. 2c). Mixed serpentine and loop findings were scored as loops, as the latter deformation seems to be the more relevant one [14].

The agreement between the three raters concerning patients’ group affiliation was almost perfect (Fleiss *k* = .92; *p* < 0.001). The transition between a normal course of the cauda equina nerve roots and a very beginning type of serpentine RNR is sometimes subtle and may lead to disagreements between the raters. In such cases the amount of straight roots on the one side of the reference stenotic level and the amount of serpentine RNR on the opposite side of the stenotic level was evaluated. If the pathologic pattern (serpentine RNR) was agreed to be prevalent (most of the roots show a serpentine shape) the case was considered as RNR+. Eighteen disagreements were reclassified in a consensus conference. Secondly, LLS and SLLS were measured. Finally, LSS-level, and LSS-grade were assessed for each patient.

### Length of lumbar spine (LLS) and segmental length of lumbar spine (SLLS) measurements

**Fig. 3:**
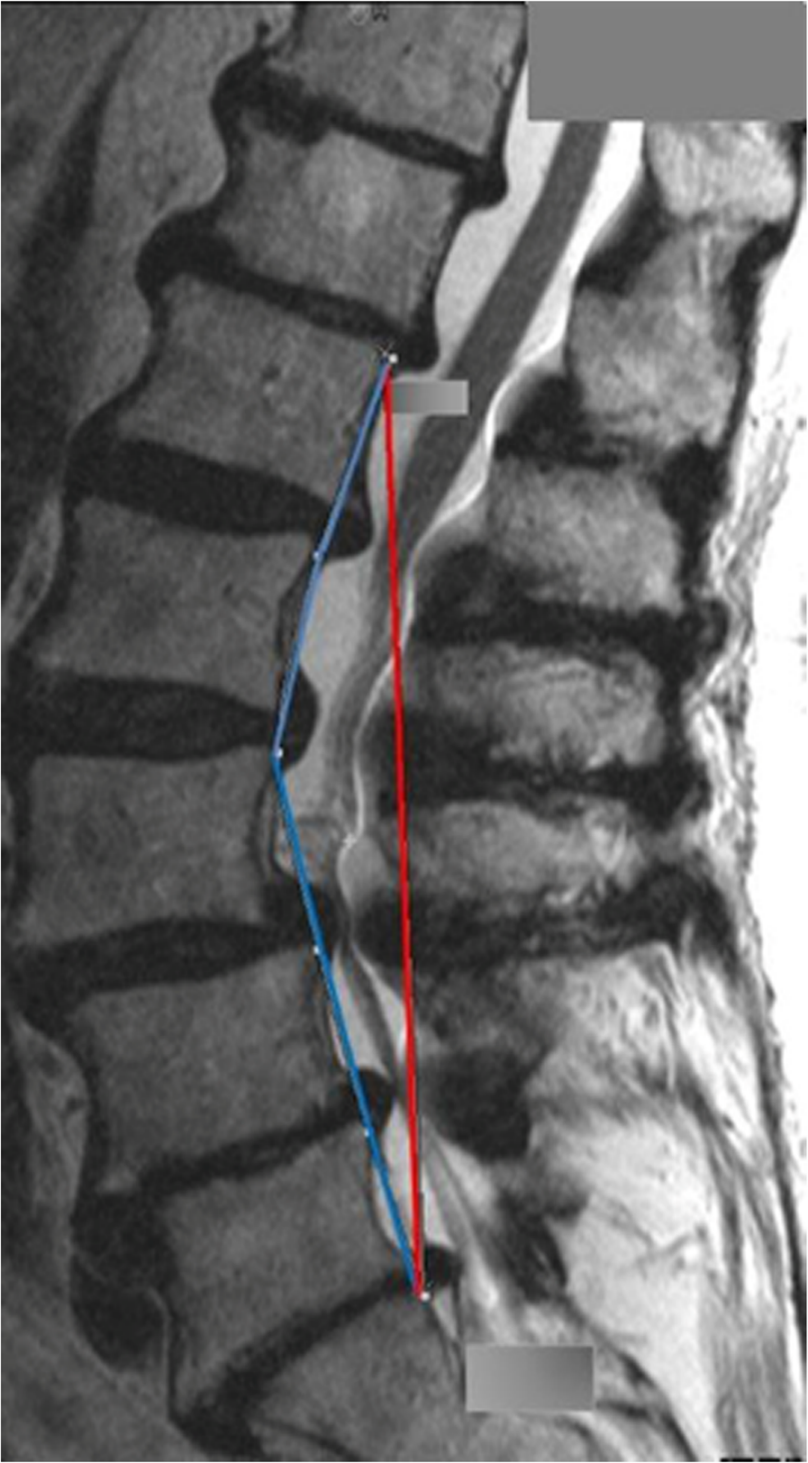
Sagittal T2-WI used for length of lumbar spine (LLS, red vector) and segmental length of lumbar spine (sLLS, blue vector) measurements

Three authors (LP, JL, TF) measured LLS and SLLS independently on the sagittal T2-weighted slice showing the midplane of the conus using the AGFA Impax 6 software (AGFA Health Care, GmbH, Bonn, Germany). For LLS measurements a straight line was drawn from the posterior-superior corner of the L1 vertebral body to the posterior-superior corner of the S1 vertebral body (Fig. 3, red line). For SLLS measurements a line was drawn from the posterior-superior corner of the L1 vertebral body to the posterior-superior corner of the L2 vertebral body. The procedure was repeated until the line reached the posterior-superior corner of the S1 vertebral body (Fig. 3, blue line). LLS and SLLS were both determined by the length of the line (mm) [15]. Inter-rater reliability for both measurements was tested previously. The estimated intraclass correlation coefficient (ICC) calculated with a two-way mixed effects model with an absolute agreement definition was .99 (95% C.I. ranging from .98 to .99) and .99 (95% C.I ranging from .97 to .99) for LLS and SLLS measurements, respectively.

### Calculation of rLLS and rSLLS

Absolute LLS and SLLS values were used to compute relative (%) rLLS and rSLLS values in relation to the patients’ body height.

### Calculation of the amount of lumbar spine alignment deviation (LSAD)

The arithmetic difference between the SLLS and LLS values of each patient was calculated as an indicator of the degree of alignment deviations of the lumbar spine (LSAD). Greater differences are caused by higher degrees of alignment deviations such as hyper-lordosis or scoliosis.

### Qualitative assessment of LSS-grade

**Fig. 4:**
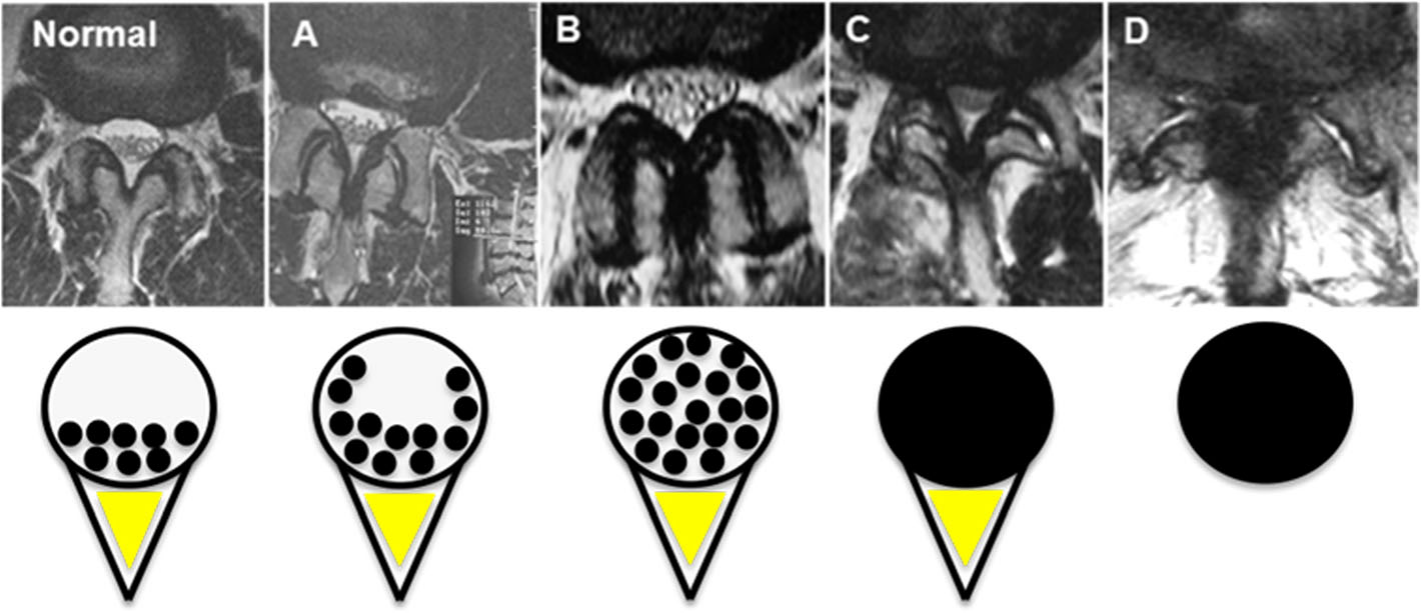
Qualitative LSS severity grade classification according to Schizas et al. (2010). *Normal*: The roots lie dorsally and occupy less than half of the dural sac area. *Grade A*: (**a**) Cerebro-spinal fluid (CSF) is clearly visible within the dural sac and the distribution of the roots is irregular. *Grade B*: (**b**) The roots are distributed through the entire cross section of the thecal sac but they can still be individualized. Some CSF is still present, giving the sac a grainy appearance. *Grade C*: (**c**) single roots cannot be recognized anymore. They appear as one gray mass that completely fills the narrowed thecal sac. There is an epidural triangle of fat (2) between the arch (1) and thecal sac. *Grade D*: (**d**) In contrast to grade C, the triangle of fat has been completely squeezed out

There is no consensus regarding the specific diagnostic criteria for lumbar spinal stenosis (LSS) based on magnetic resonance imaging (MRI) [16]. A qualitative grading system based on the root-cerebrospinal fluid (CSF) relationship was described by Schizas et al. and was found to have a prognostic value [12]. The classification includes four progressive LSS grades, with grades A and B usually responding to conservative treatment, while grades C and D often require surgical decompression [17] (Fig. 4).

Three raters independently classified the LSS-grade of the patients, and the few cases with classification discrepancies were discussed in a consensus conference.

### Quantitative assessment of LSS-level

The number of LSS-levels involved was assessed on the MRI images. A level was defined as stenotic if affected by a grade B or higher narrowing of the spinal canal. Patients were classified in three groups according to the number of stenotic levels: group 1 = one stenotic segment, group 2 = two stenotic segments, and group 3 = three or more stenotic segments involved.

### Statistical analysis

The study sample was characterized with the use of mean ± standard deviation (SD) values for continuous variables (age, BH, LLS, SLLS, rLLS, rSLLS, LSAD) and frequencies for categorical variables (gender, RNR, LSS-grade, LSS-level). Demographic data comparisons between the groups were performed with t-tests for independent samples for continuous variables. In cases in which the variable data were expressed in frequencies, chi-square tests were used to test for group dependency. Binomial logistic regressions were carried out to investigate whether the presence of RNR could be predicted by patient demographics and MRI-based measurements. Age, gender, BH, LLS, SLLS, rLLS, rSLLS, LSAD, LSS-grade and LSS-level were considered as independent variables (potential predictors). The dependent variable was group affiliation (RNR+ or RNR-). For the logistic regression LSS-grade categories A and B and LSS-levels 2 and 3 were merged due to the low number of cases in one of the categories. Single predictors were tested in the 10 models. IBM SPSS software version 21 for Macintosh (IBM Corp. Armonk, New York) was used for all statistical analyses. The 0.05 level of probability was set as the criterion for statistical significance.

## Results

### Demographic data comparisons between groups (RNR+ vs. RNR-)

RNR+ patients were 2.6 years older (*p* = 0.01) and their BH was significantly shorter by 2.9 cm (*p* = 0.01) in comparison to RNR- patients. There was no significant difference in the distribution of male and female patients in both groups (*p* = 0.3).

The mean LLS and SLLS in the RNR+ group were significantly shorter by 8.9 mm (*p* < 0.001) and 7.5 mm (*p* < 0.001), respectively. The patients in the RNR+ group had a shorter lumbar spine in relation to their BH as evidenced by their significantly smaller rLLS and rSLLS (*p* < 0.001). There were no differences between the groups concerning the amount of LSAD (*p* = 0.07) (Table 1).

**Table 1:** Demographic data

	All	RNR+	RNR-	Mean diff. (*p*-value) [95% C.I.]
Number of patients (n)	300	150	150	
Age (years)	73.5 ± 9.2	74.8 ± 8.2	72.1 ± 9.9	2.6 (*p* = 0.01) [− 4.7 to − 0.6]
Body height (cm)	173.2 ± 10.2	171.7 ± 9.9	174.6 ± 10.3	2.9 (*p* = 0.01) [0.6 to 5.2]
LLS (mm)	157.6 ± 12.6	153.2 ± 12.3	162.1 ± 11.3	8.9 (*p* < 0.001) [6.2 to 11.5]
SLLS (mm)	159.6 ± 11.8	156.1 ± 11.5	163.7 ± 11.0	7.5 (*p* < 0.001) [4.8 to 10.1]
rLLS (%)	13.4 ± 1.0	13.0 ± 0.9	13.7 ± 0.8	0.7 (*p* < 0.001) [0.5 to 0.9]
rSLLS (%)	13.6 ± 0.9	13.3 ± 0.9	13.9 ± 0.8	0.6 (*p* < 0.001) [0.4 to 0.8]
LSAD (mm)	2.6 ± 2.6	2.9 ± 2.7	2.3 ± 2.4	0.5 (*p* = 0.07) [− 1.1 to 0.05]
Gender Male (%)	196 (65.3)	94 (62.7)	102 (68.0)	_*X*_^*2*^ (1) = 0.94 (*p* = 0.3)
Female (%)	104 (34.7)	56 (37.3)	48 (32.0)	

Values are mean ± SD for age, body height, length of lumbar spine (LLS), segmental length of lumbar spine (SLLS), relative length of lumbar spine (rLLS), relative segmental length of lumbar spine (rSLLS), LSAD and frequency (%) for gender

The distribution of patients across the LSS-grade categories was significantly different between the RNR+ and RNR- groups (*p* < 0.001). In the RNR+ and RNR- groups there were 33.3 and 12.7% of patients with LSS-grade D, respectively. Patients with LSS-grade C were balanced distributed in both groups with 65.3 and 78.0% for RNR+ and RNR-, respectively. There were also significantly more patients with two and three stenotic levels in the RNR+ group (*p* < 0.001) (Table 2).

**Table 2:** Distribution of LSS-grade and LSS-level

		RNR+	RNR-	_*X*_^2^ (*P*-value)
LSS-grade	A	0	1 (0.7)	_*X*_^2^ (3) = 24.6 (*p* < 0.001)
	B	2 (1.3)	13 (8.7)	
	C	98 (65.3)	117 (78.0)	
	D	50 (33.3)	19 (12.7)	
LSS-level	1 level	102 (68.0)	127 (84.7)	_*X*_^2^ (2) = 12.5 (*p* = 0.002)
	2 levels	42 (28.0)	22 (14.7)	
	3 levels	6 (4.0)	1 (0.7)	

Values are frequencies (%)

### Predictors of RNRs

Gender was not a significant predictor of RNRs (*p* = 0.3). The likelihood of RNR+ (Odds Ratio) increased 1.06 times as patients’ age increased by two years (*p* = 0.02). A 3 cm decrease in BH increased the chance of RNR+ group membership by 1.09 times (*p* = 0.01).

As LLS and SLLS decreased by 5 mm, the likelihood of RNR+ increased by 1.36 and 1.34 times, respectively (*p* < 0.001). A 1% decrease in rLLS and rSLLS increased the odds of RNR+ by 2.26 and 2.17 times, respectively (*p* < 0.001).

The amount of LSAD was not a significant RNR predictor (*p* = 0.07).

**Fig. 5:**
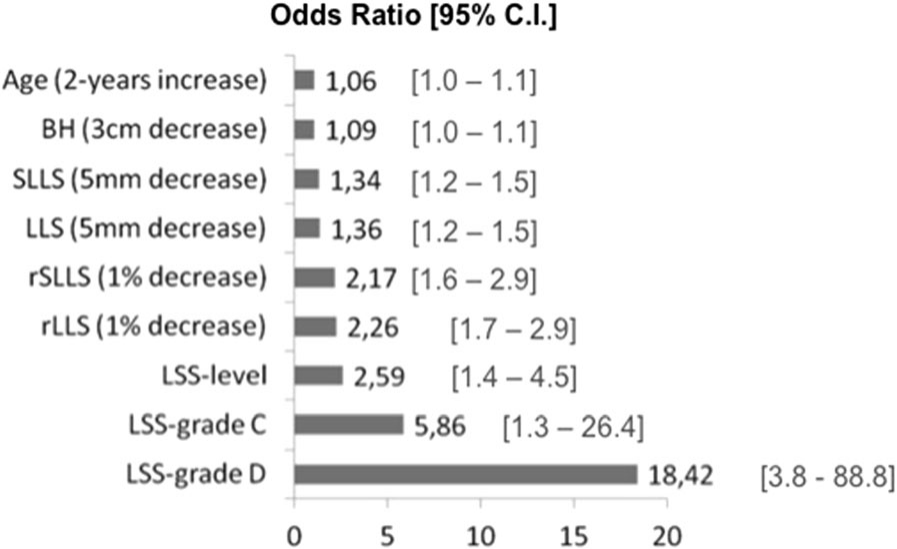
Significant predictors of RNR with the estimated odds ratios

In patients with LSS-levels 2 and 3 the odds of RNR+ increased 2.59 times compared to patients with LSS-level 1 (*p* = 0.001) (Fig. 5).

Patients with LSS-grade C were 5.86 times more likely to show RNR signs (*p* = 0.02), and LSS-grade D had a 18.4 times increased chance of RNR+ (*p* < 0.001) when comparing with patients affected by LSS-grade A and B (Table 3).

**Table 3:** Results of the binomial logistic regression models

Model	Independent variables included	Negelkerke R^2^	Odds ratio (OR)	[95% C.I.]	*p*-value
1	Gender (Female)	.00	1.26	[0.78 to 2.03]	*p* = 0.3
2	Age^(1)^	.02	1.06	[1.01 to 1.12]	*p* = 0.01
3	Body height^(2)^	.02	1.09	[1.01 to 1.16]	*p* = 0.01
4	LLS^(3)^	.16	1.36	[1.23 to 1.52]	*p* < 0.001
5	SLLS^(4)^	.13	1.34	[1.20 to 1.50]	*p* < 0.001
6	rLLS^(5)^	.17	2.26	[1.76 to 2.95]	*p* < 0.001
7	rSLLS^(6)^	.14	2.17	[1.63 to 2.90]	*p* < 0.001
8	LSAD	.01	1.08	[0.99 to 1.19]	*p* = 0.07
9	LSS-level^(7)^	.05	2.59	[1.48 to 4.55]	*p* = 0.001
10	LSS-grade	.11			*p* < 0.001
	grade C^(8)^		5.86	[1.30 to 26.42]	*p* = 0.02
	grade D^(9)^		18.42	[3.82 to 88.8]	*p* < 0.001

OR for group membership in RNR+, *LSS* Lumbar Spinal Stenosis, *LLS* Length of Lumbar Spine

^(1)^OR for a 2 years increase in patients age

^(2)^OR for a 3 cm decrease in body height

^(3)^OR for a 5 mm decrease in LLS

^(4)^OR for a 5 mm decrease in SLLS

^(5)^OR for a 1% decrease in rLLS

^(6)^OR for a 1% decrease in rSLLS

^(7)^OR for patients classified as LSS-level 2 + 3; reference were patients classified as LSS-level 1

^(8)^OR for patients classified as LSS-grade C; reference were patients classified as LSS-grade A + B

^(9)^OR for patients classified as LSS-grade D, reference were patients classified as LSS-grade A + B

## Discussion

The reported prevalence rates of RNR among LSS patients vary and range from 15% [7] to 45.5% [18], with the majority of studies reporting RNR prevalence rates around 40% [2, 3, 19]. Although some studies have evidenced the negative prognostic effect of RNR on postsurgical recovery of LSS-patients [2, 8, 14], no work previously investigated the potential weight of patient demographics and MRI-based measurements in predicting RNRs in LSS-patients. The main findings of the present study are as follows:

Patient-related and MRI-based measurements can predict the presence of RNR in LSS patients. The strongest predictors of RNR were LSS severity grade D, OR = 18.4, 95% C.I. [3.8 to 88.8], LSS severity grade C, OR = 5.8, 95% C.I. [1.3 to 26.4], LSS-level, OR = 2.5, 95% C.I. [1.4 to 4.5] and rLLS, OR = 2.2, 95% C.I. [1.7 to 2.9].

In the present study the patients in the RNR+ group were 2.6 years older (*p* = 0.01). This finding is in line with previous observations [6, 7, 9, 19] and a recent published meta-analysis [20]. In the literature the mean age difference between patients with or without RNR varies and ranges from 7.8 years [6] to 13.8 years [9]. Comparable mean ages between patients with or without RNR signs were reported in only two former studies [3, 21].

The mean BH of RNR+ patients was shorter by 2.9 cm (*p* = 0.01) and their LLS and SLLS were also significantly shorter by 8.9 mm and 7.5 mm, respectively (*p* < 0.001). Interesting is also the fact that the rLLS in RNR+ was shorter by 0.7% in relation to patients BH in comparison to RNR- patients (*p* < 0.001). The same was observed for rSLLS (mean diff. 0.6%, *p* < 0.0001). In view of these findings, the question is whether an aging-related degeneration of the lumbar spine with an absolute and a relative shortening of LLS and consequently a reduction in the length of the spinal canal plays a role in the pathogenesis of RNR?

The pathogenesis of RNR is still unclear. Suzuki et al. suggested that the squeezing force from the constricted spinal canal acting on the nerve roots causes the elongation and is the origin of RNR [9]. This explanation has not been questioned ever since. In the present study we have searched for significant predictors of RNR among patient-related factors. To the authors’ best knowledge, no previous study measured and compared the LLS, SLLS, rLLS and rSLLS in patients with or without RNR.

In the mid eighties Tsuji et al. [18] raised the hypothesis that age-dependent shortening of the lumbar spine may be connected to the pathogenesis of RNR. This assumption was never investigated since then but the present results seem to confirm it. rLLS and rSLLS were both significant predictors of RNR+ (*p* < 0.001). A 1% reduction in rLLS increased the odds of RNR+ by 2.26 times. The rLLS was the third strongest patient-related predictor of RNR.

Our results are also consistent with the explanation suggested by Suzuki et al. [9], since compression of the cauda equina nerve roots (LSS-grade) was the strongest RNR+ predictor. LLS-grade C increased the odds of RNR+ by 5.8 times, 95% C.I. [1.3 to 26.4], and LLS-grade D increased the chance of RNR+ by 18.42 times, 95% C.I. [3.8 to 88.8]. Our results also identified additional important factors in the pathogenesis of RNR, like the number of stenotic levels involved and the rLLS or rSLLS (Fig. 5).

When considering LSS severity, it is interesting to note that patients with LLS-grade C were similarly distributed in RNR+ (65.3%) and RNR- (78.0%) groups. Furthermore, 12.7% of RNR- patients were classified as LSS-grade D. How can the high percentage of RNR- patients (77%) that did not develop RNRs although affected by LSS-grade C or D be explained? Age-related LLS shrinking could make the difference. To clarify this question further investigation is needed.

Based on the present results the lumbar spine could be considered as the discal-osseous-ligamentous “container” of the cauda equina nerve roots. The nerve roots could be considered as the “content”. The container shrinks due to aging-related degenerative changes in the lumbar spine, but at the same time the roots of the cauda equina, fixed between conus medullaris and intraforaminal ganglia, keep their length. It sounds plausible that a progressive mismatch between container and content could origin a relative “over-length” of the cauda nerve roots. These can develop a serpentine-like shape at the beginning and a loop-like course in a further stage. The mismatch seems to ground on individual changes in the relationship between “container” and “content” and is evidenced by a smaller rLLS in relation to patients’ body height.

There was a significant difference in the distribution of LSS-levels between RNR+ and RNR- patients (*p* = 0.002) (Table 2). Thirty-two percent of RNR+ but only 15.4% of RNR- patients had two or more stenotic levels. Multi-segmental stenosis seems to interfere more with the natural course of the cauda nerve roots than single-level stenosis. This result confirms the one reported by Hur et al. [3]. It also confirms the importance of the “total amount” of compression in the pathogenesis of RNR that could be quantified as sum of LSS-grade and LSS-levels.

Poureisa et al. [7] reported that age (OR = 1.0, *p* = 0.01), the location of the stenosis (OR = 2.5, *p* < 0.001) and the presence of a sharp intracanal protuberance at the stenotic level (OR = 7.2, *p* < 0.001) were significantly and independently associated with RNR. Chen et al. [2] recently demonstrated that greater lumbar lordosis angles in extension and in neutral position, as well as a greater overall range of motion, were significantly associated with RNR. These results reinforce the assumption that RNR in LSS patients are caused by multiple factors and not only by compression.

Degenerative spondylolisthesis of grade higher than “1” according to the Meyerding [22] classification was an exclusion criterion in the present study. This probably explains why the amount of lumbar spine alignment deviation (LSAD) was not different between both groups (*p* = 0.07) and was not a significant RNR predictor. In contrast, Savarese et al. included patients with any degree of spondylolisthesis and reported that vertebral slip increased the prevalence of RNR by 3.5 times [19]. They also concluded that spondylolisthesis is an independent risk factor for RNR. For this reason we have decided, in the planning stage of the present work, to exclude patients diagnosed with LSS secondary to spondylolisthesis from the sample.

Due to the retrospective study design the number of potential predictors was restricted to the available data. This is a study limitation. There were no available data on clinical scores. A future study with a prospective study design should consider the assessment of clinical scores and functional data, such as the preoperative walking distance.

## Conclusions

Patient-related factors were different between patients with or without RNR signs. Multiple factors are associated with the presence of RNR in LSS patients. Severe stenosis grade D or grade C, two or more stenotic levels and a shorter relative length of the lumbar spine were strong determinants of RNR.

## Abbreviations

BH: Body height
CSF: Cerebro spinal fluid
LLS: Length of lumbar spine
LSAD: Lumbar spine alignment deviation
LSS: Lumbar spinal stenosis
MRI: Magnetic resonance imaging
OR: Odds ratio
rLLS: Relative length of lumbar spine
RNR-: No evidence of RNR
RNR +: Evidence of RNR.
RNRs: Redundant nerve roots
rSLLS: Relative segmental length of lumbar spine
SLLS: Segmental length of lumbar spine
